# Plasmonic Nanostructures for Photothermal Conversion

**DOI:** 10.1002/smsc.202000055

**Published:** 2021-01-18

**Authors:** Jinxing Chen, Zuyang Ye, Fan Yang, Yadong Yin

**Affiliations:** ^1^ Department of Chemistry University of California Riverside CA 92521 USA; ^2^ Jiangsu Key Laboratory for Carbon-Based Functional Materials and Devices Institute of Functional Nano and Soft Materials (FUNSOM) Soochow University Suzhou Jiangsu 215123 P. R. China

**Keywords:** light to heat, photoactuation, photothermal conversion, photothermal therapy, plasmonic, solar energy harvesting

## Abstract

The nonradiative conversion of light to heat by plasmonic nanostructures, the so‐called plasmonic photothermal effect, has attracted enormous attention due to their widespread potential applications. Herein, the perspectives on the design and preparation of plasmonic nanostructures for light to heat or photothermal conversion are provided. The general principle of plasmonic photothermal conversion is first introduced, and then, the strategies for improving efficiency are discussed, which is the focus of this field. Then, five typical application types are used, including solar energy harvesting, photothermal actuation, photothermal therapy, laser‐induced color printing, and high‐temperature photothermal devices, to elucidate how to tailor the nanomaterials to meet the requirements of these specific applications. In addition to the photothermal effect, other unique physical and chemical properties are coupled to further explore the application scenarios of plasmonic photothermal materials. Finally, a summary and the perspectives on the directions that may lead to the future development of this exciting field are also given.

## Graphical abstract

The plasmonic photothermal property of metallic nanostructures can effectively convert light into heat, thereby inducing unique light‐controllable nanoscale heating. The composition and structure of these nanoscale photothermal converters can be rationally designed to achieve high performance in various intriguing applications, including solar energy harvesting, photothermal actuation, photothermal therapy, laser‐induced color printing, and high‐temperature photothermal devices.

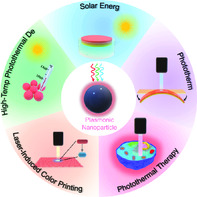

## 1 Introduction

Plasmonic metal nanostructures have significant applications in a wide range of areas such as energy conversion,^[^ ^1^, ^2^ ^]^ sensing,^[^ ^3^, ^4^ ^]^ and biomedicines due to their unique optical properties.^[^ ^5^, ^6^ ^]^ When the energy of incident light matches the resonance frequency of the free electrons, the metal nanoparticles exhibit strong localized surface plasmon resonance (LSPR).^[^ ^7^, ^8^ ^]^ Similar to the phonon, which is the quantized expression of the collective oscillation motion of atoms in the crystal, the surface plasmon is the quasi‐particle that represents the quantization of plasma oscillation due to the interaction between conduction electrons and electromagnetic fields.

**Figure 1:**
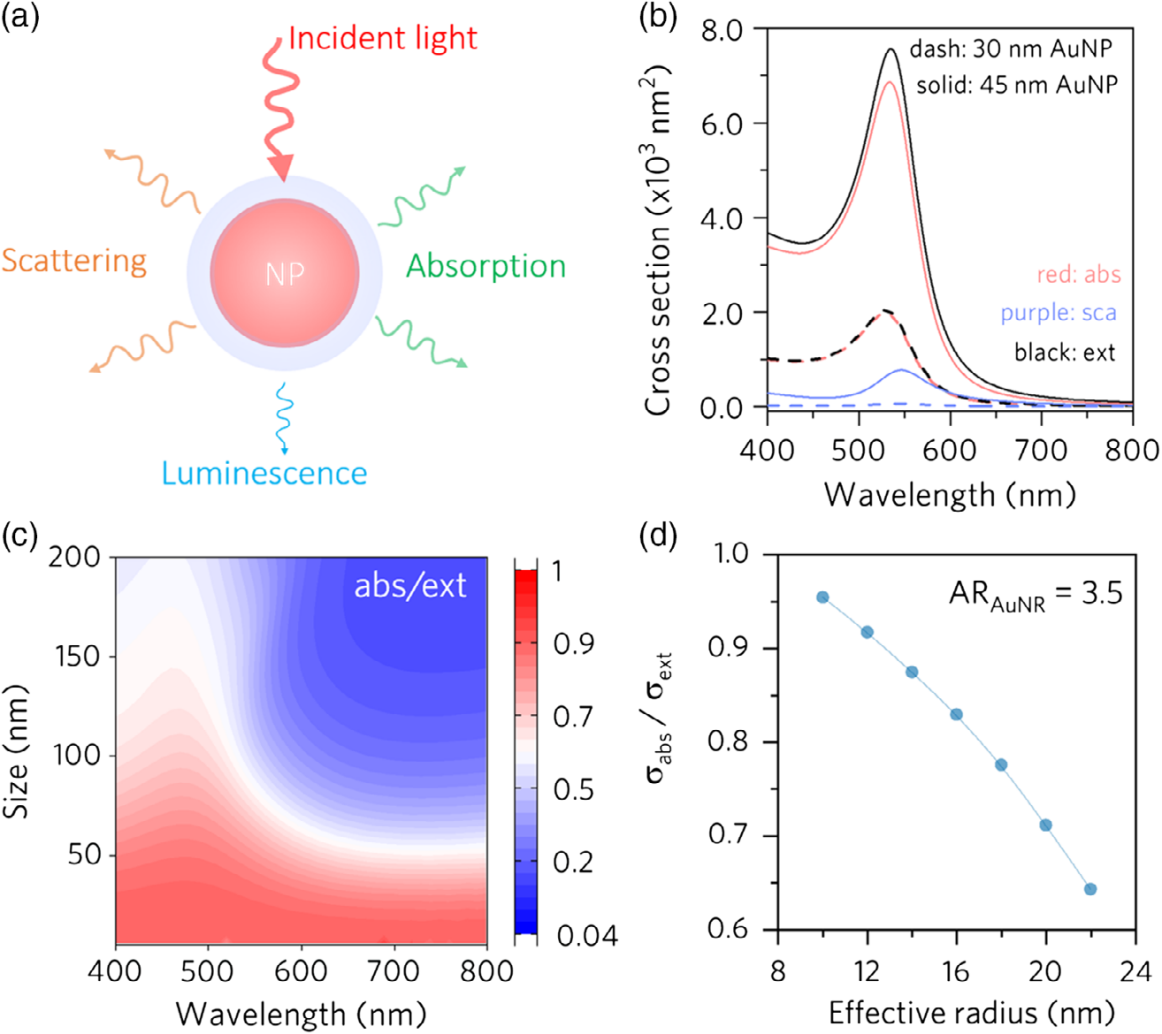
Plasmonic photothermal conversion. a) Schematic illustration of the different modes of interaction between the light and a plasmonic nanoparticle. b) Simulated absorption, scattering, and extinction cross sections of Au nanoparticles with different sizes. c) Intensity plot of absorption efficiency as a function of diameter and wavelength for Au nanoparticles in water. d) Dependence of the absorption efficiency of AuNRs as a function of their effective radius for a fixed aspect ratio of 3.5. The effective radius was defined as *r*_eff_ = (3V/4π)^1/3^, where *V* is the volume of AuNRs.

The excitation of plasmon oscillations in a metal nanoparticle induces a strong absorption and scattering of the incident light (**Figure** **1**a), depending on materials composition, dimension, and morphology, as well as the dielectric constant of surroundings. The total number of photons interacting with a metal nanoparticle can be expressed by its extinction cross section (*σ*_ext_), which is given by the sum of the absorption (*σ*_abs_) and scattering (*σ*_sca_) cross sections (*σ*_ext_ = *σ*_abs_ + *σ*_sca_). The energy absorbed by plasmonic nanoparticles can then be released by either the re‐emission of photons (luminescence) or the generation of phonons (heat). Previous works have concluded that the quantum yield of luminescence for plasmonic nanoparticles is below 1%; therefore, it can be assumed that all the absorbed energy is transformed to heat.^[^ ^9^ ^]^ Notably, the light absorption cross section of metal nanoparticles greatly exceeds their physical cross sections at the resonance wavelength, endowing them with strong light‐to‐heat conversion ability.

The photothermal conversion process can be understood as follows. Upon light illumination, the plasmonic nanostructures can absorb light of a certain wavelength range, which induces LSPR and causes the electrons to oscillate. The electrons jump to an excited energy state, followed by the electron–electron scattering at subpicosecond timescales,^[^ ^10^ ^]^ thereby leading to the redistribution of hot electrons. Subsequently, heat is transferred to the metal lattice through the electron–phonon coupling and further dissipates from the lattice to the surrounding medium within nanoseconds by phonon–phonon coupling.^[^ ^11^, ^12^, ^13^, ^14^ ^]^

Unlike the conventional heating methods, these nanoscale metal heaters allow the heat to be localized in a submicrometer space, which arouses intense interest in practical applications. Much effort has been made to investigate the photothermal conversion property of plasmonic nanoparticles, and various nanostructures have been proposed to enhance the efficiency.

In this perspective, we aim to discuss the structure–activity relationship between plasmonic nanostructure and their photothermal conversion effect and establish the design principles of the metal nanostructures for various applications. We begin by introducing the photothermal properties of plasmonic nanostructures and then discuss the general strategies for minimizing the scattering cross section and enhancing the photothermal conversion efficiency. Our focus is then shifted to discussing specific applications of the plasmonic photothermal nanomaterials, i.e., solar energy harvesting, photothermal actuation, photothermal therapy (PTT), laser‐induced color printing, and high‐temperature photothermal devices. We will touch on the performance and deficiency of the current designs in the relevant fields, followed by possible methods for enhancing the photothermal efficiency and enabling new functionalities. Finally, we put forward some personal views on the challenges and prospects of plasmonic photothermal materials.

## 2 Photothermal Efficiency in Plasmonic Nanostructures

Regardless of the application, the pursuit of high light‐to‐heat efficiency has been the focus of research as it is directly related to the device performance. It, therefore, requires an in‐depth understanding of the light–nanostructure interaction. According to the Mie–Gans theory, the cross sections of absorption (*σ*_abs_), scattering (*σ*_sca_), and total extinction (*σ*_ext_) can be described by the following equations.^[^ ^15^, ^16^ ^]^

(1) $$\sigma_{\text{abs}}=\frac{2\pi }{3\lambda }\varepsilon_{m}^{3/2}V\sum_{i}\frac{\varepsilon_{2}/{\left(n^{\left(i\right)}\right)}^{2}}{{\left(\varepsilon_{1}+\left[\left(1-n^{\left(i\right)}\right)/n^{\left(i\right)}\right]\varepsilon_{m}\right)}^{2}+\varepsilon_{2}^{2}}$$

(2) $$\sigma_{\text{sca}}=\frac{8\pi^{3}}{9\lambda^{4}}\varepsilon_{m}^{2}V^{2}\sum_{i}\frac{\left[{\left(\varepsilon_{1}-\varepsilon_{m}\right)}^{2}+\varepsilon_{2}^{2}\right]/{\left(n^{\left(i\right)}\right)}^{2}}{{\left(\varepsilon_{1}+\left[\left(1-n^{\left(i\right)}\right)/n^{\left(i\right)}\right]\varepsilon_{m}\right)}^{2}+\varepsilon_{2}^{2}}$$

(3) $$\sigma_{\text{ext}}=\sigma_{\text{sca}}+\sigma_{\text{abs}}$$

where *λ* is the light wavelength, *ε*_m_ is the dielectric constant of the surrounding, *ε* is the dielectric constant of the metal deﬁned by *ε* = *ε*_1_ + *iε*_2_ (*ε*_1_ and *ε*_2_ are the real and imaginary parts of the dielectric constant, respectively), *n* ^(*i*)^ is the depolarization factor, and *V* is the unit volume of the nanoparticle. Furthermore, *n* ^(*i*)^ is expressed as follows

(4) $$n^{\left(a\right)}=\frac{1}{R^{2}-1}[\frac{R}{2\sqrt{R^{2}-1}}\ln\frac{R+\sqrt{R^{2}-1}}{R-\sqrt{R^{2}-1}}-1]$$

(5) $$n^{\left(b\right)}=n^{\left(c\right)}=(1-n^{\left(a\right)})/2$$

where *a*, *b*, and *c* indicate geometric factors (i.e., the three axes) of the nanoparticle, and *R* is the aspect ratio. Importantly, the LSPR occurs when the *ε*_1_ is equal to −(1 − *n* ^(*i*)^)×*ε*_m_/*n* ^(*i*)^.

Equation (1) and (2) suggest that the size (volume) of the nanoparticles affects the cross sections of both absorption and scattering but at different magnitudes, with *σ*_sca_ (∝ *V* ^2^) being more sensitive to the change in *V* than *σ*_abs_ (∝ *V*). Therefore, as the size of nanoparticles increases, the scattering cross section will gradually dominate the extinction cross section (Figure 1b). As it determines the photothermal conversion efficiency, we calculated the absorption efficiency (*σ*_abs_/*σ*_ext_) for Au nanospheres with a broad range of diameters. As shown in Figure 1c, in general, smaller Au nanoparticles exhibit higher absorption efficiency in the entire visible spectrum (400–800 nm), suggesting their advantage in the effective conversion of light energy to heat.

Au nanostructures with more complicated geometries than spheres, such as Au nanorods (AuNRs) and nanoshells, also exhibit dimensional dependence of absorption and scattering cross sections. Among various non‐spherical metal nanoparticles, AuNRs have attracted particular attention, as they can be readily synthesized with precisely controlled aspect ratio.^[^ ^17^, ^18^, ^19^ ^]^ The size dependence of AuNRs with a fixed aspect ratio of 3.5 on their absorption efficiency is simulated and shown in Figure 1 d. As the volume of AuNRs increases, the absorption becomes less relevant, which further confirms that the small size of plasmonic nanostructures is a sufficient condition to ensure the high photothermal conversion efficiency. The calculated values have been proved by the experimental measurement of ~90% efficiency for small AuNRs (38×10 nm).^[^ ^20^, ^21^ ^]^ However, one potential issue of these small AuNRs is their poor thermal stability, which brings many challenges to practical applications.^[^ ^22^, ^23^, ^24^, ^25^, ^26^ ^]^

In addition to the size and shape effects, Equation (1) and (2) suggest that the dielectric constant of the surrounding medium (*ε*_m_) also significantly influences the optical properties of plasmonic nanostructures. For example, for Au nanoparticles with a diameter of 80 nm, a numerical study has shown that the scattering cross section increases rapidly when the dielectric constant increases from 1.0 to 2.0.^[^ ^27^ ^]^ At the same time, the absorption cross section exhibits only a slight change, thereby reducing the absorption efficiency. In practice, however, it is difficult to tune the plasmonic resonance by manipulating the dielectric constant of the surrounding medium, because the media, such as the tissue fluid in PTT, typically remains unchanged in a specific system and process. Nevertheless, a transition layer can be introduced between the nanoparticle and the surrounding to establish an increasing gradient of refractive index (*n*_nanoparticle_ < *n*_transition layer_ < *n*_surrounding_) to increase the absorption efficiency.^[^ ^28^, ^29^ ^]^ Conversely, placing a transition layer that does not conform to the increasing trend may result in reduced absorption.

## 3 Plasmonic Nanostructures Tailored for Specific Applications

While improving photothermal conversion efficiency is of universal significance for plasmonic photothermal applications, additional requirements should be considered during the design of nanoparticle structures for specific cases. To this end, we review the use of plasmonic photothermal conversion in several typical scenarios to highlight the key factors that need to be considered, including solar energy harvesting, photothermal actuation, PTT, laser‐induced color printing, and high‐temperature photothermal devices.

### 3.1 Solar Energy Harvesting

With the continuous process optimization and improvement of energy conversion efficiency, solar energy is increasingly regarded as an alternative source to replace fossil fuels for electricity generation.^[^ ^30^, ^31^ ^]^ The key challenge of solar‐thermal conversion by plasmonic nanomaterials is to broaden the absorption band to cover the whole solar spectrum. The absorption wavelength and intensity of plasmon excitations strongly depend on not only their intrinsic material properties, including composition, size, and shape, but also their assembly configuration.

The composition effect on the plasmonic property can be illustrated by Equation ((1), (2))–(3), mainly reflected by the dielectric constant of materials (*ε*_1_ and *ε*_2_). Typically, spherical Al and Pd nanoparticles are plasmonically active in the ultraviolet range, whereas Ag, Au, and Cu nanoparticles mainly work in the wavelength range of 400–650 nm. Other nonmetal plasmonic nanostructures, such as Cu_2−*x*_S and MoO_*x*_, generally show absorption from ultraviolet to near‐IR (NIR) range. For example, sub‐stoichiometric MoO_3−*x*_ nanobelts with high oxygen vacancy density have been synthesized through a surface‐ligand protected reduction strategy. These nanostructures show tunable plasmonic absorption in a wide wavelength range from 200 to 2500 nm.^[^ ^32^ ^]^

**Figure 2:**
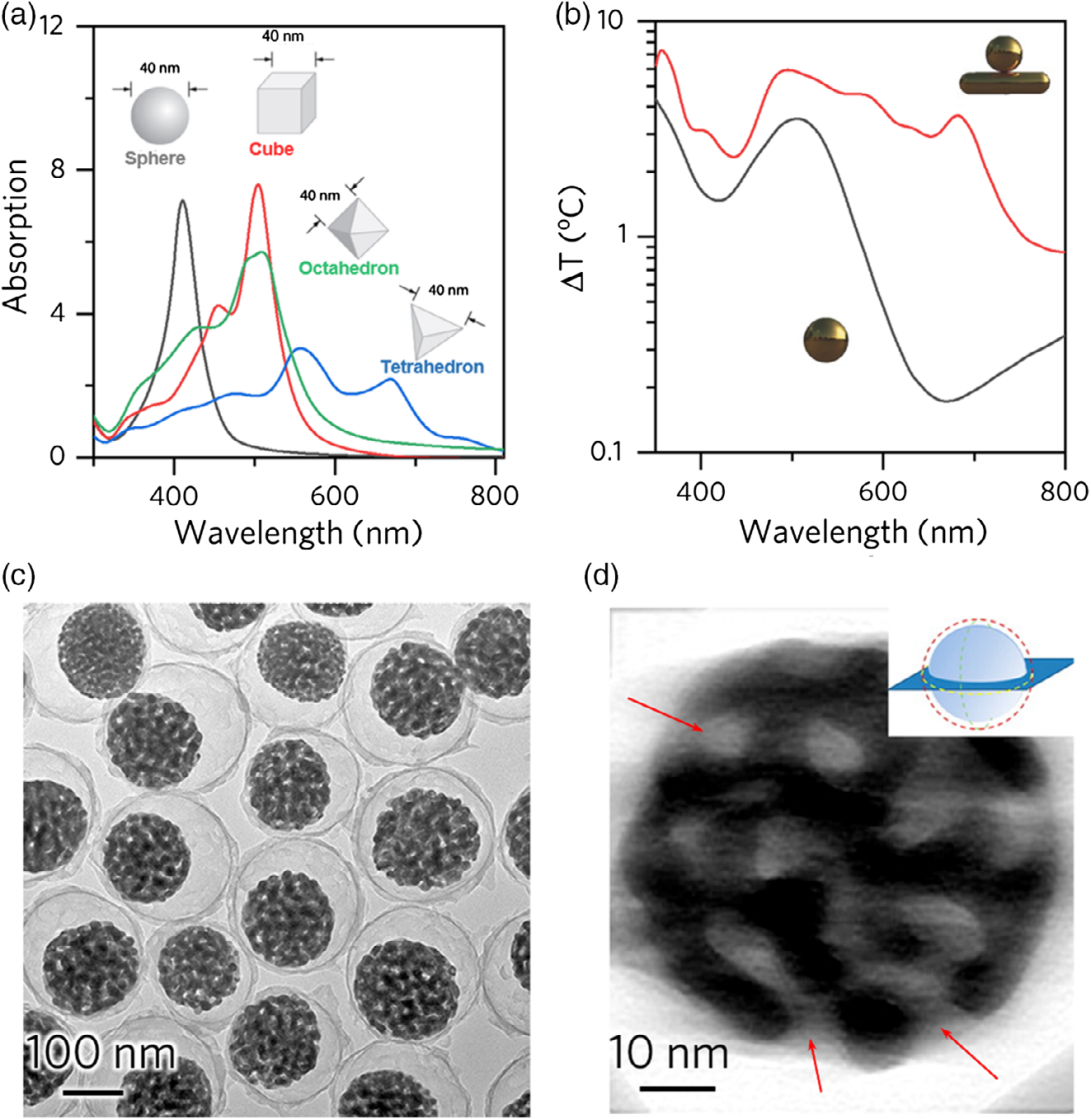
Plasmonic nanostructures with broadband absorption. a) The dependence of absorption spectra of plasmonic materials on the shape. Adapted with permission.^[^ ^35^ ^]^ Copyright 2006, American Chemical Society. b) Simulated temperature profiles of Au nanostructures. Adapted with permission.^[^ ^36^ ^]^ Copyright 2017, Wiley‐VCH. c) Transmission electron microscopy (TEM) image of porous Au–Ag @void@SiO_2_ nanoparticles. d) Virtual slice from the reconstructed tomogram of an individual porous Au–Ag nanoparticle. Adapted with permission.^[^ ^45^ ^]^ Copyright 2016, American Chemical Society.

Xia and co‐workers calculated the absorption spectra of Ag nanostructures with the same size but various shapes (**Figure** **2**a).^[^ ^33^, ^34^, ^35^ ^]^ Ag nanostructures with high symmetry, such as spheres and cubes, exhibit strong absorption peaks. On the contrary, the polyhedron particles with low symmetry have much broader but weak absorption peaks with multiple resonances. An intuitive strategy for broadening the absorption band is to directly mix nanostructures with various shapes. But, this method requires multiple parallel syntheses and careful control over the ratio of different nanostructures, making it impractical for most large‐scale applications.

An ideal solar absorber is one material capable of broadband absorption. For example, Han and co‐workers synthesized asymmetric Au nanostructures by growing a Au nanoparticle on each pre‐existing AuNR to effectively harvest the incident light by taking advantage of the broadened optical resonance due to the randomly distributed particles on AuNRs (Figure 2b).^[^ ^36^ ^]^ When the as‐synthesized Au nanostructures were deposited on a Si substrate, a layer with a thickness of only 10.2 μm could achieve an average broadband absorption of 98.43% with a peak value of 99.7%. In a demonstration of driving solar steam generation, these Au nanostructures exhibited a solar‐to‐vapor efficiency of 76% under 1 sun illumination.^[^ ^37^ ^]^

Plasmonic nanostructures with many built‐in “hotspots” have also been utilized for realizing broadband absorption.^[^ ^38^, ^39^ ^]^ Many efforts have been made to produce such hotspots, for example, by constructing Au/Ag nanoparticles with sharp tips, rough surface, and inter‐ or intraparticle nanogaps.^[^ ^40^, ^41^, ^42^, ^43^, ^44^ ^]^ However, the density of hotspots in conventional plasmonic nanostructures is typically low, and it is challenging to meet the requirements for solar applications. As demonstrated by our group, dealloying is one promising method to fabricate porous Au–Ag alloy nanoparticles with built‐in high‐density hotspots (Figure 2c).^[^ ^45^ ^]^ They were synthesized by etching less‐stable Ag from completely alloyed Au–Ag nanospheres (Figure 2d), resulting in a strong absorption from 300 to 1800 nm.

**Figure 3:**
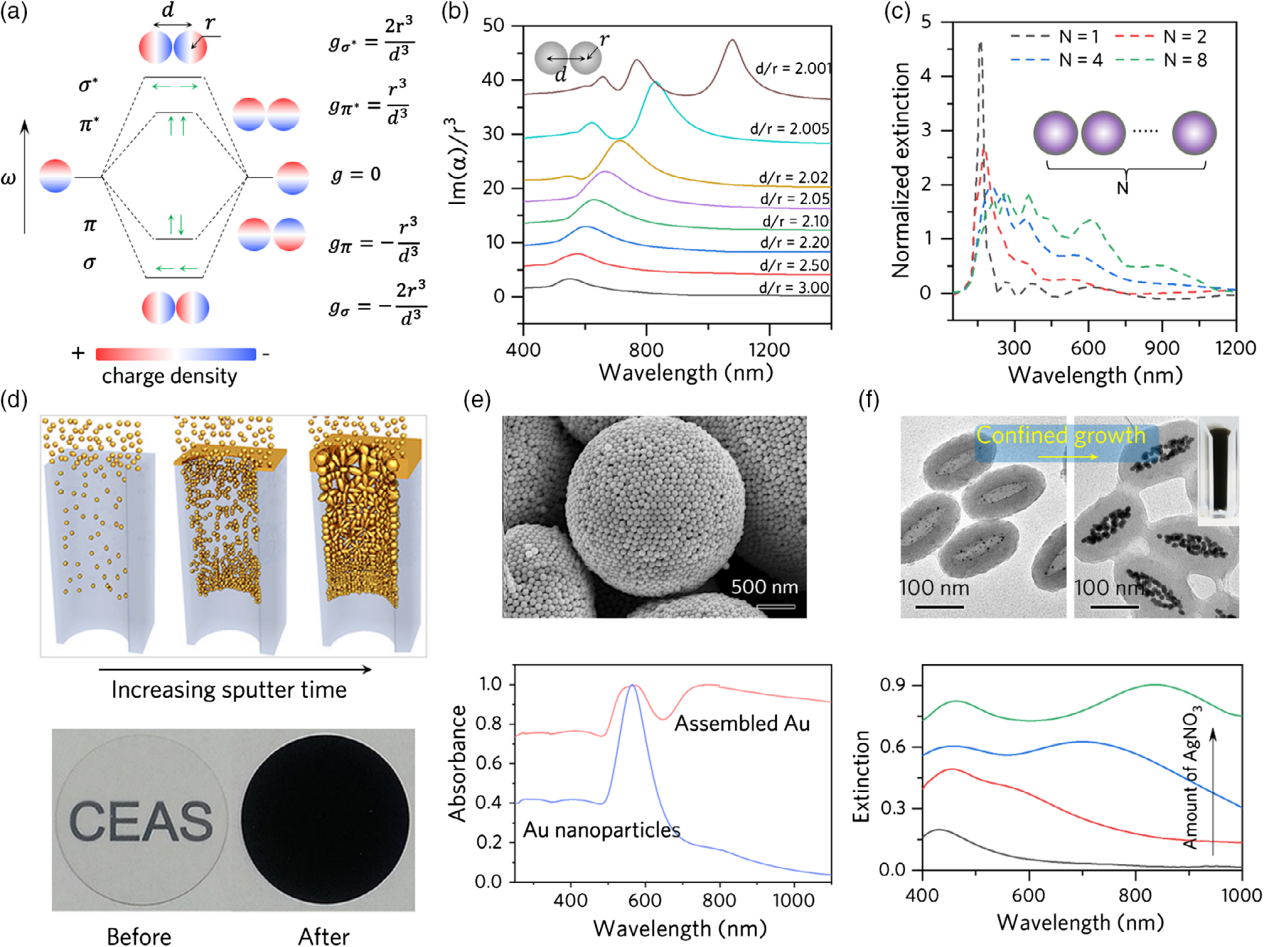
Boosting and broadening absorption spectrum by plasmonic coupling effect. a) Energy hybridization in a plasmonic dimer system. Adapted with permission.^[^ ^50^ ^]^ Copyright 2008, Royal Society of Chemistry. b) Absorption spectra of the Au nanospheres dimer with different interparticle distances. Adapted with permission.^[^ ^51^ ^]^ Copyright 2006, Optical Society of America. c) Dependence of calculated normalized extinction cross section (σ_ext_/σ_geo_) of Al nanoparticles on the particle number *N*. Adapted with permission.^[^ ^57^ ^]^ Copyright 2016, Springer Nature. d) Self‐assembly of Au nanoparticles on nanoporous templates. The transparent template is changed to black. Adapted with permission.^[^ ^58^ ^]^ Copyright 2016, American Association for the Advancement of Science. e) Scanning electron microscopy (SEM) image and absorption spectrum of the self‐assembled hollow Au microspheres. Adapted with permission.^[^ ^62^ ^]^ Copyright 2015, Wiley‐VCH. f) TEM images of templates before and after seeded growth (upper). UV–vis–NIR extinction spectra of the colloidal dispersions at different growth stages (bottom). Adapted with permission.^[^ ^63^ ^]^ Copyright 2015, Wiley‐VCH.

Another widely used strategy to broaden the absorption band and promote electron oscillations of plasmonic nanostructures is to induce the interparticle coupling of LSPR, which can occur when two or more plasmonic nanoparticles come sufficiently close.^[^ ^46^, ^47^, ^48^, ^49^ ^]^ For simplicity, we use a plasmonic dimer as an example to describe the typical plasmon hybridization process.^[^ ^50^ ^]^ As shown in **Figure** **3**a, a dimer exhibits two dipolar resonance modes, which are determined by the polarization direction. For polarization parallel to the dimer axis (longitudinal), the parallel arrangement of dipoles results in a low‐energy “bonding” mode, whereas the tail‐to‐tail arrangement of dipoles leads to a high‐energy “anti‐bonding” mode. The bonding mode is bright, and the anti‐bonding mode is dark, as only the former has a finite total dipole moment. When polarization and dimer axis are perpendicular to each other (transverse), the parallel dipoles produce a bright high‐energy mode, and the anti‐parallel dipoles generate a dark low‐energy mode. The plasmon hybridization could further lead to band splitting and broadening. Figure 3b shows the simulated spectral evolution of a dimer composed of two identical Au nanospheres with a diameter of 120 nm and an interparticle distance of *d*.^[^ ^51^ ^]^ When *d* is equal to 3*r*, the interaction between two particles is weak, and the single‐particle dipole mode dominates the spectrum. The bonding mode then shows a redshift as interparticle distance decreases. With the further decrease in *d* to near touch, a higher multipolar bonding mode, such as the bonding quadrupolar mode, appears in the absorption spectrum. While the broadening of the LSPR peak for a dimer is limited, plasmonic coupling involving many interacting nanoparticles can sufficiently increase absorption bandwidth.^[^ ^52^, ^53^, ^54^, ^55^, ^56^ ^]^ As shown in the simulated results in Figure 3c, the plasmon hybridization of a linear assembly of aluminum (Al) nanoparticles can induce a drastic redshift of the resonant wavelength toward visible and NIR regime when more nanoparticles are included.^[^ ^57^ ^]^

The above‐mentioned theoretical analysis suggests a promising strategy for enhancing photothermal conversion through structural engineering of the plasmonic assemblies. Zhu et al. prepared a plasmonic absorber by depositing Au nanoparticles on a nanoporous alumina template (Figure 3d). The Au nanoparticles down in the pores were distributed randomly, enabling a high density of hybridized LSPR to fully absorb sunlight. Meanwhile, the Au deposited on the surface forms a metallic film, which suppresses the back reflection of photons. As a result, the film can enable an average absorbance of ≈99% across the visible to IR spectrum (400 nm to 10 μm).^[^ ^58^ ^]^ When used for interfacial solar steam generation, the film exhibited a high solar‐to‐vapor efficiency (≈90%) under sunlight irradiation (400 mW cm^−2^).

Plasmonic colloidosomes composed of close‐packed plasmonic nanoparticles, generally fabricated by the self‐assembly method,^[^ ^59^, ^60^, ^61^ ^]^ may also serve as an alternative candidate for generating strong plasmonic coupling. For example, Liu et al. fabricated black‐colored 3D Au colloidosomes using an emulsion‐templating approach (Figure 3e).^[^ ^62^ ^]^ This strategy is universal and suitable for metallic nanoparticles of all shapes, such as Au nanooctahedra and Au–Ag heterogeneous nanorods. However, the overall dimension of the plasmonic colloidosomes is limited to the micrometer scale, which may cause a large interfacial scattering.

We have recently reported a space‐confined seeded growth strategy for the preparation of black Ag nanostructures.^[^ ^63^ ^]^ By controlling the growth of seeds pre‐fixed in a narrow space, plasmonic coupling with high density can be achieved (Figure 3f). Notably, the individual Ag nanoparticles and their overall assemblages are small, minimizing the scattering effect and maximizing the conversion of light to heat. With the use of black Ag nanostructures as the light absorber, the interfacial steam generation device achieved a photothermal efficiency of over 95%.

### 3.2 Photothermal Actuation

Actuation responding to external stimuli is a feature commonly found in nature. Many plants and animals can alter their structures in response to various environmental stimuli.^[^ ^64^, ^65^, ^66^, ^67^ ^]^ Inspired by nature, novel functional materials have been developed to fabricate actuators that can exhibit pre‐designed shape deformations triggered by stimuli, such as temperature,^[^ ^68^, ^69^ ^]^ humidity,^[^ ^70^, ^71^ ^]^ light,^[^ ^72^ ^]^ and electricity.^[^ ^73^ ^]^ With the elaborate structural design, these smart actuators may find numerous applications ranging from soft robotics^[^ ^20^, ^74^ ^]^ to biomedicines.^[^ ^75^, ^76^ ^]^

While most actuators rely on pneumatic or electric systems tethered to external pumps or circuits, light‐driven actuators have attracted significant attention because of their unique feature of remote control.^[^ ^77^ ^]^ To convert photon energy to mechanical deformations, photoactive agents, such as photothermal materials (e.g., nanoparticles or organic dyes) and photochemical switches (e.g., azobenzene‐based molecules), are needed. Photons emitted by a light source and absorbed by a photoactive agent may produce mechanical motions by 1) photothermal expansion;^[^ ^78^, ^79^ ^]^ 2) conformational change;^[^ ^80^, ^81^ ^]^ or 3) phase transition (e.g., glass transition of shape‐memory materials,^[^ ^82^, ^83^ ^]^ nematic‐isotropic transition of liquid crystals,^[^ ^84^, ^85^ ^]^ and hydrophilic–hydrophobic transition of hydrogels.^[^ ^86^, ^87^ ^]^)

**Figure 4:**
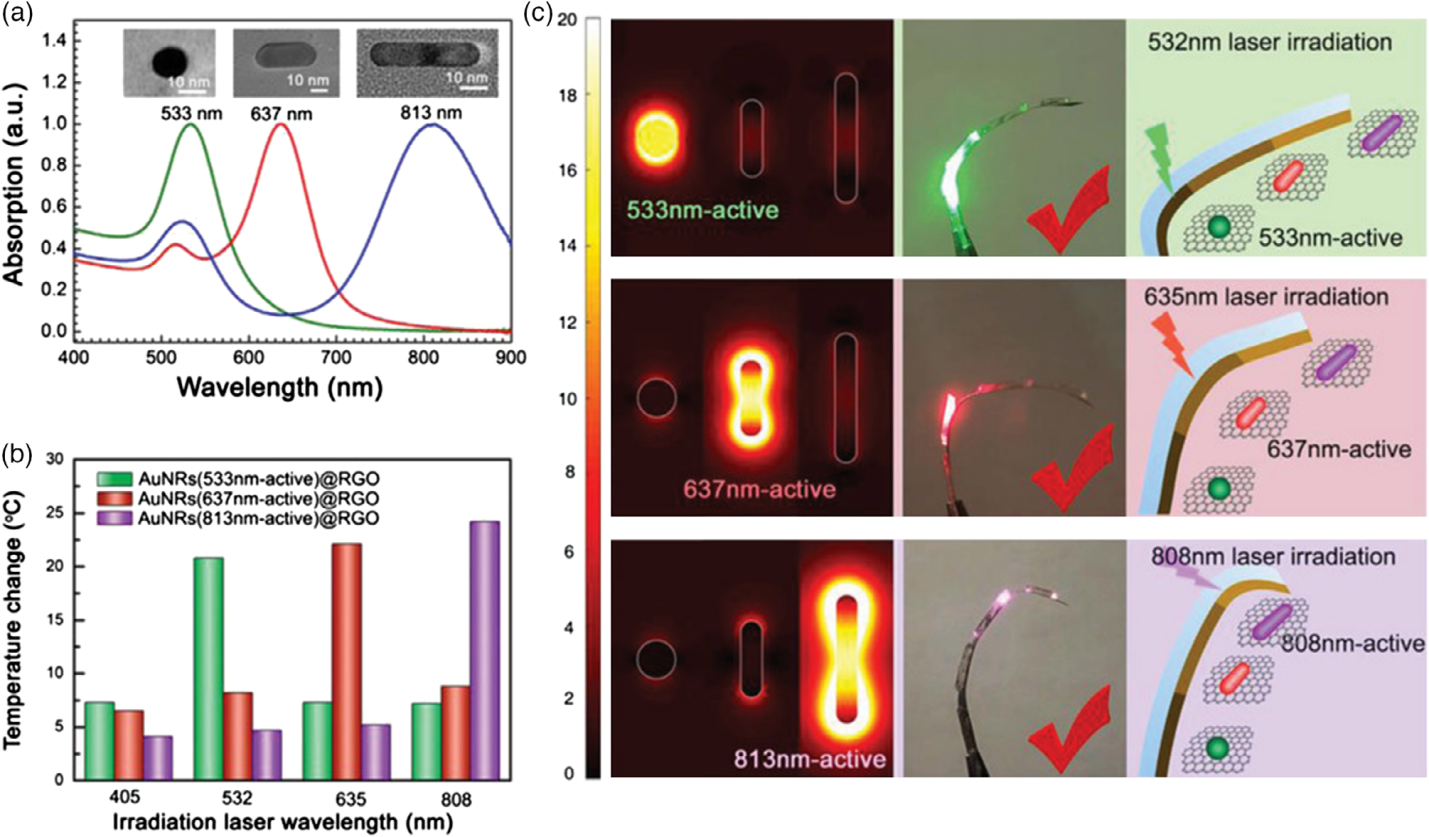
Wavelength‐selective properties of photothermal actuators. a) Normalized absorption spectra of AuNRs with different aspect ratios. b) Maximum temperature variation of bimorph actuators prepared using different AuNRs (with central absorption peaks of 533, 637, or 813 nm) under the stimulation of lasers (≈50 mW cm^−2^) with the wavelengths of 405, 532, 635, or 808 nm. c) Simulation of electric field enhancement of three different AuNRs and the corresponding response of ribbons with three different bimorphs active to the lasers of 532, 635, or 808 nm wavelength. Adapted with permission.^[^ ^90^ ^]^ Copyright 2018, Wiley‐VCH.

Most light‐assisted actuators can only show a single motion due to the lack of wavelength selectivity of the light absorbers.^[^ ^88^ ^]^ The plasmonic nanoparticles can selectively absorb incident light in a specific wavelength range and convert it to localized heat. Therefore, to achieve various actuation modes by manipulating light, one would expect the plasmonic materials to have the geometrical anisotropy, bringing about two or more plasmonic peaks that can be activated by light of different wavelengths.^[^ ^89^ ^]^ For example, if one can take advantage of AuNRs’ transverse and longitudinal plasmonic excitation at two distinctive wavelengths, it is promising to fabricate actuators with dual response. Moreover, the concept of wavelength‐selective photothermal heating can be extended to more than two wavelengths by utilizing many AuNRs with different aspect ratios. Sun and co‐workers fabricated finger‐like actuators containing AuNRs with resonance peaks at 533, 637, and 808 nm at different joints (**Figure** **4**).^[^ ^90^ ^]^ Due to the wavelength‐dependent photothermal effect of AuNRs, the joints will be heated only when the laser wavelength matched the absorption band of AuNRs, thereby triggering the bending of different knuckles. By integrating various wavelength‐responsive actuators, many complex actuation modes can be realized to mimic the flexibility and versatility of natural systems without the spatial control of the light source.

**Figure 5:**
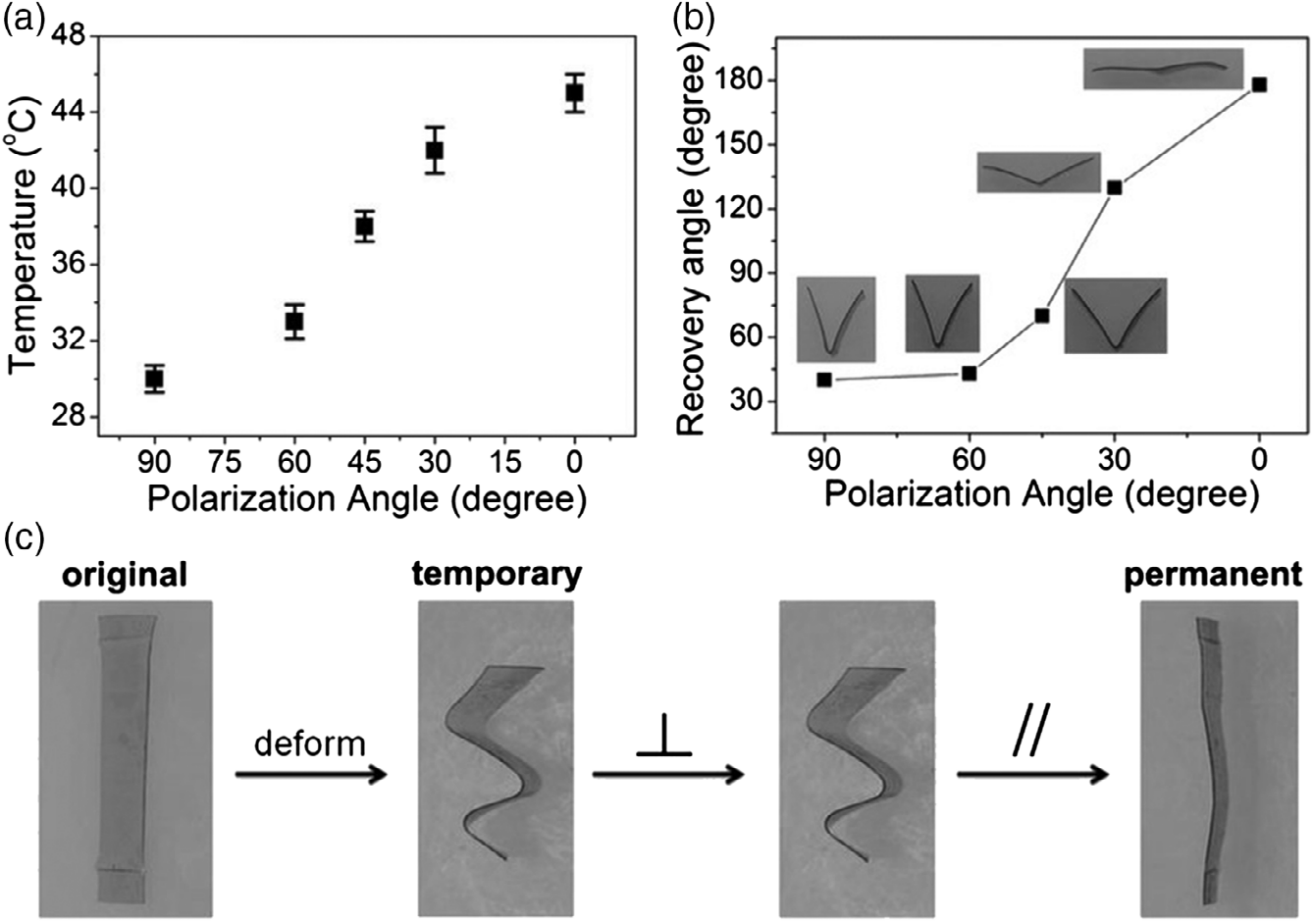
Polarization selective properties of photothermal actuators. a) Temperature rise in PVA/AuNR (0.02 wt%) film exposed to the 785 nm laser with a fixed laser power of 220 mW cm^−2^ at various polarization angles. b) Recovery angle versus laser polarization angle (1 min exposure), showing controllable shape recovery extent using light polarization. c) Photographs showing the polarization‐dependent shape recovery: exposing first the folds to laser with perpendicular polarization for 2 min gives rise to no shape recovery; subsequently, 10 s exposure to laser with parallel polarization results in full shape recovery. Adapted with permission.^[^ ^93^ ^]^ Copyright 2013, Wiley‐VCH.

Wavelength‐selective actuators usually require multiple light sources to provide different wavelength inputs, which is unfavorable for miniaturization. A unique feature of plasmonic nanostructures with anisotropic morphology is the polarization‐dependent photothermal heating. To absorb the incident light at the LSPR wavelength, the orientation of anisotropic nanostructures should also match the polarization of light to give the maximum absorbance. However, typically colloidal particles are randomly distributed, showing no polarization‐selective photothermal behaviors. By applying a force during or after incorporating particles into polymer matrixes, the alignment of particles can be achieved. For example, aligning AuNRs can be achieved in polymer films, giving rise to the enhancement of photothermal conversion efficiency when the polarization direction of light overlaps the nanorods’ long axis.^[^ ^91^, ^92^ ^]^ Maity et al. obtained self‐aligned AuNRs in the poly(ethylene oxide) (PEO) matrix utilizing the electrostatic and viscoelastic forces during the electrospinning of the fibers.^[^ ^92^ ^]^ The as‐fabricated, AuNRs‐embedded nanofibrous mat exhibited polarization‐sensitive photothermal heating. The fibers can undergo phase changes upon the illumination of polarized light parallel to fiber alignment while remaining unchanged under perpendicularly polarized light. Besides, Zhao and co‐workers aligned AuNRs by stretching the embedding poly(vinyl alcohol) (PVA) film and demonstrated a shape‐memory behavior that depended on light polarization (**Figure** **5**).^[^ ^93^ ^]^ The longitudinal peak intensity of AuNRs can be tuned continuously by varying the polarization direction of the incident light relative to the stretching direction of the film, leading to differences in the rise of local temperature upon light exposure (Figure 5a). Therefore, by only changing the laser polarization, temporary‐to‐permanent shape recovery of the composite may occur to varying degrees (Figure 5b,c). The manipulation of light polarization, thus, represents another useful strategy for the fabrication of photothermal actuators.

**Figure 6:**
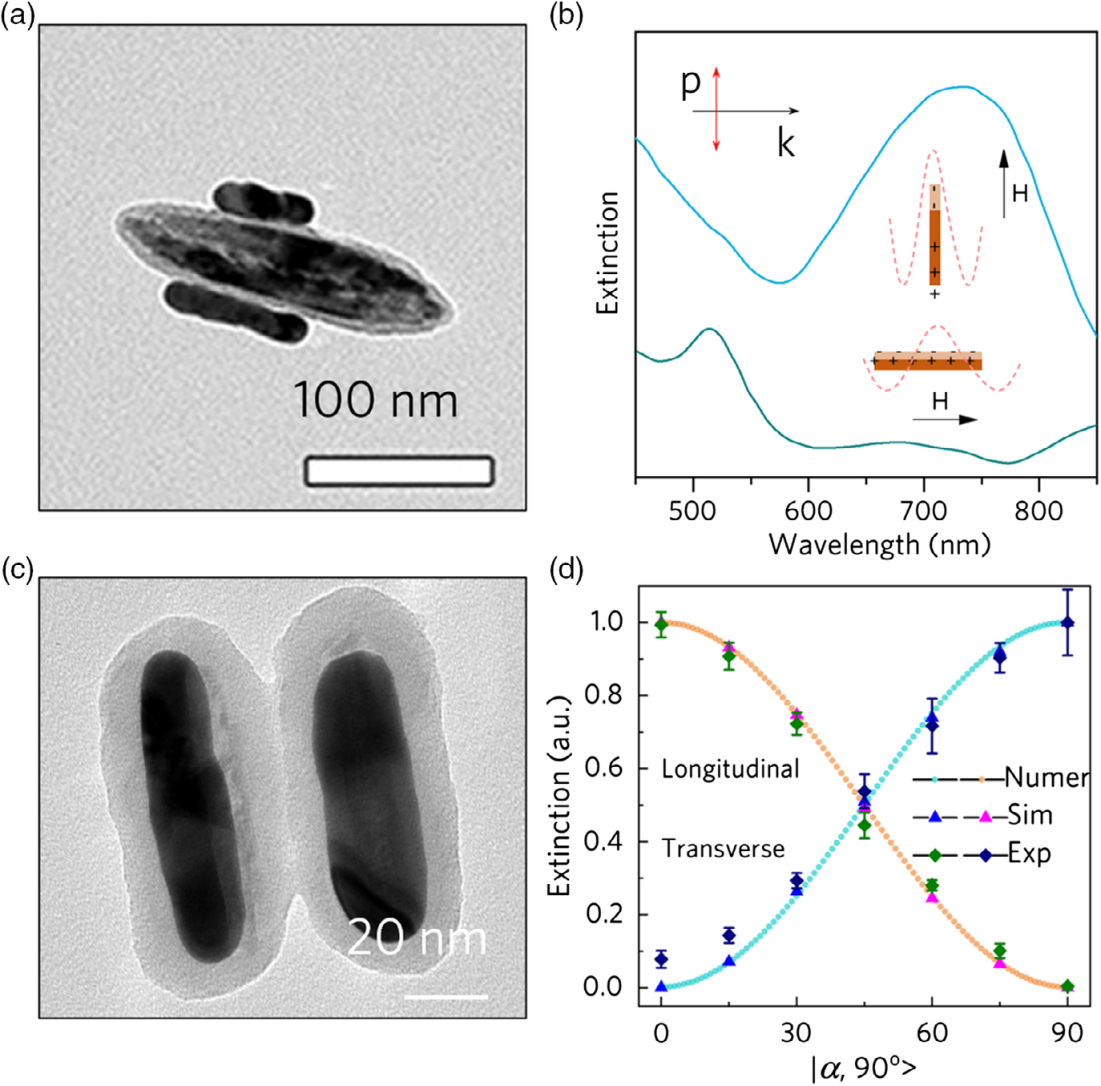
Optical tunability of magnetic/plasmonic hybrid nanostructures. a) TEM image of the Au attached Fe_3_O_4_ nanorod. b) Spectra of dispersion of the hybrid nanostructures under a magnetic field with its direction perpendicular or parallel to incident light. Adapted with permission.^[^ ^97^ ^]^ Copyright 2005, American Chemical Society. c) TEM image showing hybrid nanorods synthesized by the space‐confined seeded growth method. d) Orientation‐dependent plasmonic excitation of AuNRs. Adapted with permission.^[^ ^98^ ^]^ Copyright 2020, Springer Nature.

The selective actuation by light polarization has not been widely explored due to two main challenges: 1) the lack of appropriate methods for effective alignment of anisotropic nanostructures; and 2) the aggregation of particles in the polymer matrix, resulting in imperfect alignment.^[^ ^91^ ^]^ We have recently explored magnetic means to maneuver the alignment of nanostructures.^[^ ^94^, ^95^, ^96^ ^]^ In our early effort, we reported magnetic tuning of the plasmonic property of AuNRs by attaching AuNRs onto magnetic Fe_3_O_4_ nanorods and then controlling their orientation using an external field (**Figure** **6**a).^[^ ^97^ ^]^ When the magnetic field was along the propagation direction of the incident light, only the transverse mode can be excited. When the field was turned perpendicular to the incidence direction, the longitudinal mode will be excited. The effect became more obvious under polarized light. Under an external magnetic field, the hybrid nanorods could be aligned either parallel or perpendicular to the polarization direction of the incident light. When the magnetic field was parallel to the polarization of light, the longitudinal mode would dominate (blue curve in Figure 6b); while if the magnetic field was perpendicular to the polarization, the longitudinal mode would be suppressed (green curve in Figure 6b). This work showed the feasibility of selectively controlling the activation mode of anisotropic materials. However, the as‐synthesized hybrid nanorods tend to aggregate at a high concentration, hindering the application in photothermal actuation.

Later, we have addressed this issue by developing a space‐confined seeded growth method to synthesize well‐defined magnetic‐plasmonic hybrid nanostructures.^[^ ^98^ ^]^ Anisotropic seeded growth of Au was initiated in a rod‐shaped void space created in a polymer shell by coating a Fe_3_O_4_ nanorod, producing a pair of parallelly aligned Au/Fe_3_O_4_ nanorods wrapped in each shell, as shown in Figure 6c. The resulting hybrid nanorods exhibited high structural and colloidal stability, allowing convenient control of the longitudinal mode of the AuNRs using an external magnetic field even at a high particle concentration (Figure 6d). This method provides an accessible way to control the size and morphology of plasmonic structures and to couple the plasmonic and magnetic anisotropy, promising for polarization‐modulated photothermal actuation. As an additional advantage, the polymer surface of the hybrid nanostructures can be easily modified to enhance the compatibility with the matrix and avoid aggregation.

More recently, we have developed a freestanding, multidirectional robot by simply modulating the polarization and on–off switch of a single laser.^[^ ^99^ ^]^ This design is enabled by the conﬁned growth of hybrid Fe_3_O_4_/Ag nanorods with a polymer shell. The hybrid nanorods can be collectively aligned along speciﬁc directions in a solid ﬁlm by applying a magnetic ﬁeld. Incorporating the nanorods into photocurable polymers enables the construction of actuators whose mechanical bending can be controlled by switching laser polarization. By simply combining the polarization‐dependent properties of hybrid nanorods into a bipedal robot, we have demonstrated the four basic movements of a robot.

### 3.3 Photothermal Therapy

**Figure 7:**
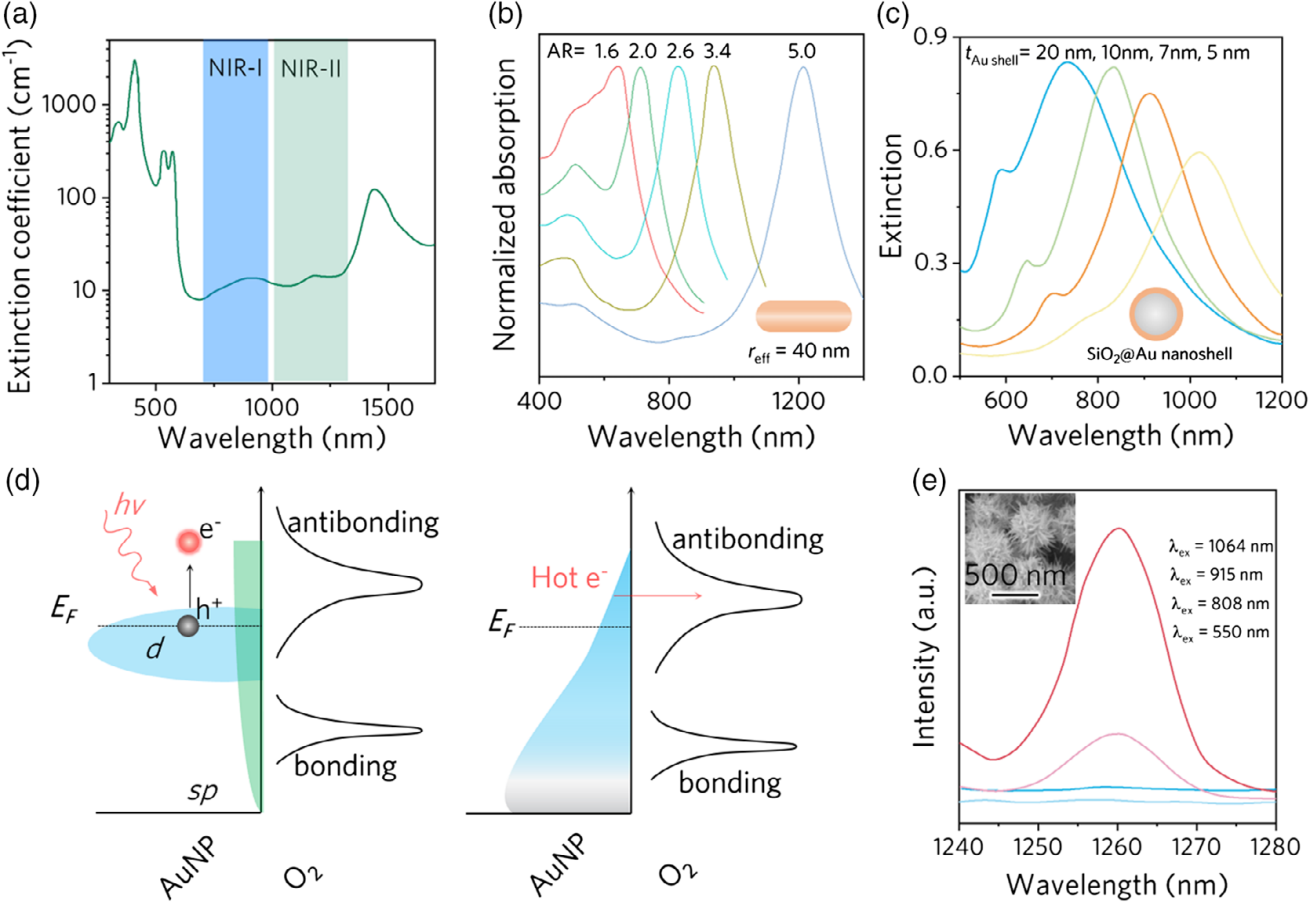
Typical plasmonic nanomaterials for PTT. a) Extinction coefficient of a representative tissue. b) The absorption cross section of AuNRs with varying aspect ratios but fixing the effective radius of 40 nm. Adapted with permission.^[^ ^107^ ^]^ Copyright 2005, American Chemical Society. c) The absorption cross section of SiO_2_@Au nanoparticles with different shell thicknesses. Adapted with permission.^[^ ^109^ ^]^ Copyright 2007, Elsevier. d) A schematic depicting the initial step in hot‐electron‐induced activation of O_2_ by transferring a hot electron into the anti‐bonding orbital of the molecule. Adapted with permission.^[^ ^110^ ^]^ Copyright 2014, American Chemical Society. e) Singlet oxygen phosphorescence emission spectra sensitized by Au nanoechinus at 550, 808, 915, and 1064 nm excitation wavelengths. Inset: SEM image of Au nanoechinus. Adapted with permission.^[^ ^124^ ^]^ Copyright 2014, Wiley‐VCH.

PTT refers to efforts to convert light to heat to treat various medical conditions such as tumors. Traditionally, laser‐induced thermal ablation was regarded as a non‐reliable and low‐efficient technique due to the need for high‐power laser irradiation. High light intensity causes damage to healthy tissues because of the strong extinction abilities of normal human tissues (**Figure** **7**a). Nevertheless, PTT is nowadays receiving considerable attention, because it has been possible to lower the required light intensity by localizing heat generation using light‐absorbing nanoparticles at a diseased region.^[^ ^100^, ^101^, ^102^, ^103^ ^]^ Light absorption by healthy tissues can be further reduced using a laser with its wavelength in the biological windows where tissues have a low extinction coefficient. According to the different absorption bands, there are two biological windows of NIR‐I (700–980 nm) and NIR‐II (1000–1400 nm). Working in the biological windows reduces the non‐selective heating of healthy tissue, thereby allowing deep tissue treatments. Thus, it is desirable to synthesize nanostructures with light absorption in the NIR window.

With the absorption wavelength tunable from visible to IR, anisotropic plasmonic nanostructures are suitable candidates for bioapplications.^[^ ^104^, ^105^, ^106^ ^]^ As shown in Figure 7b, increasing the aspect ratio of AuNRs gradually shifts the longitudinal absorption peak from visible to NIR‐II window.^[^ ^107^ ^]^ Another representative example of Au nanostructures with a peak located at the NIR windows is Au nanoshell,^[^ ^108^, ^109^, ^110^, ^111^ ^]^ and its LSPR band can be tuned by either changing the overall size, or the diameter of hollow space, or the shell thickness. Figure 7c presents the absorption cross section of Au nanoshells, each of which consists of a SiO_2_ core (60 nm in diameter) and an Au nanoshell with varying thicknesses (5–20 nm). As the thickness decreases, the geometrical anisotropy increases, leading to the redshift of the resonant peak.

Plasmonic nanostructures hold great promise for PTT applications, as they enable effective, non‐invasive, and highly targeted cancer therapy. As many reviews have been made to summarize the development in this research field,^[^ ^102^, ^109^, ^112^, ^113^, ^114^ ^]^ we will not include a detailed discussion on this topic here. Nevertheless, we want to point out the requirements that the light‐absorbing nanomaterials have to meet to achieve efficient PTT: 1) large absorption cross sections in the biological windows; 2) easy post‐modifications; 3) good stability in biological setting for long‐term circulation; and 4) low cytotoxicity to ensure human health. The photothermal ablation of tumor cells is a promising approach for the treatment of local tumors. Yet, it is difficult to completely eradicate large tumors due to residual tumor mass at the treatment margins. A combination of multiple strategies may be considered to address this issue. For example, developing an integrated nanoparticle‐based platform of PTT and chemotherapy would elicit robust anti‐tumor responses. It can be achieved using hollow or porous plasmonic structures (e.g., Au nanobox, porous Au nanospheres) as the photothermal agents. Upon light illumination, the generated heat can directly kill local cancer and promote the release of loaded drugs to eliminate metastatic cancer.

Besides the photothermal effect, the light absorbed by plasmonic nanostructures in tumor cells can also generate reactive oxygen species (ROS), which would induce cancer cell apoptosis.^[^ ^115^, ^116^, ^117^, ^118^ ^]^ This process is called photodynamic therapy (PDT). Obviously, both PTT and PDT utilize the nonradiative decay of LSPR and rely on the light‐absorption properties of the nanostructures.^[^ ^119^ ^]^ As designing plasmonic nanostructures to enhance PTT will also promote their PDT performance, we include a brief discussion of PDT in the following part. As a star material, AuNRs can generate a very high ROS concentration under two‐photon excitation at 808 nm, and their efficiency is much higher than commercial photosensitizers, such as indocyanine green.^[^ ^120^ ^]^ In addition, the Au nanostructures can resist photobleaching and enzymatic degradation, which, in combination with their large absorption cross sections, makes them a promising candidate agent for PDT.

The photochemical mechanism of PDT by plasmonic nanostructures is shown in Figure 7d. The excited surface plasmons first relax into hot electrons. The hot electrons in the high‐energy tail are then transferred to the surrounding oxygen molecules to populate the 2π* anti‐bonding O–O orbital and produce a transient negative $O_{2}^{-\cdot }$. Then, the relaxation of $O_{2}^{-\cdot }$ releases an electron back to the plasmonic surface, and the electron back‐transfer process results in a spin‐flip of O_2_, forming ^1^O_2_.^[^ ^112^, ^121^, ^122^ ^]^ Due to the high‐energy state of ^1^O_2_, it is very reactive to surrounding matters, such as enzymes or DNA, causing destruction to cancer cells.^[^ ^123^ ^]^ Current studies have shown that ROS is usually generated by sharp tips and defects of plasmonic nanostructures. For instance, Hwang and co‐workers have prepared multi‐branched Au nanoechinus for activated PDT and PTT in the NIR‐II window (Figure 7e).^[^ ^124^ ^]^ Upon excitation at the first (915 nm) and second (1064 nm) biological windows, phosphorescence emission of ^1^O_2_ was clearly observed (≈1267 nm). In contrast, no ^1^O_2_ signal was observed with 808 and 550 nm light excitation. The mechanism of spectral‐selective excitation, however, still needs further clarification.

As for the PDT, the light dose‐ and oxygen‐dependent therapeutic effect is still expected to be further improved, especially in the hypoxia condition of the solid tumor site. The development of new photosensitizers with excitation light in the biological window is particularly urgent, because organic photosensitizers usually have low quantum efficiency in the biological window. This can be improved using plasmonic nanostructures, because their absorption band can be tailored and shifted to the NIR‐II window. However, the mechanism of PDT in plasmonic nanostructures needs to be explored further. For example, the shape‐dependent and spectral‐selective (Figure 7e) excitation of ROS is interesting, but more efforts should be made to gain an in‐depth understanding of the fundamentals in PDT.

Another critical aspect of nanomaterials relating to bioapplications is their biocompatibility. Many plasmonic structures, especially for anisotropic ones, such as AuNRs, nanoplates, and nanobipyramids, were synthesized using halide‐based ligands, which would cause severe cytotoxicity. Therefore, it remains an open challenge for material chemists to produce anisotropic plasmonic nanostructures with low cytotoxicity. As an effort in this direction, we have synthesized Au nanoplates with tunable absorption windows by H_2_O_2_ reduction with the assistance of polyvinylpyrrolidone (PVP).^[^ ^125^ ^]^ The PVP‐capped Au nanoplates were found to offer much‐improved biocompatibility than those prepared using halide‐based surfactants.

**Figure 8:**
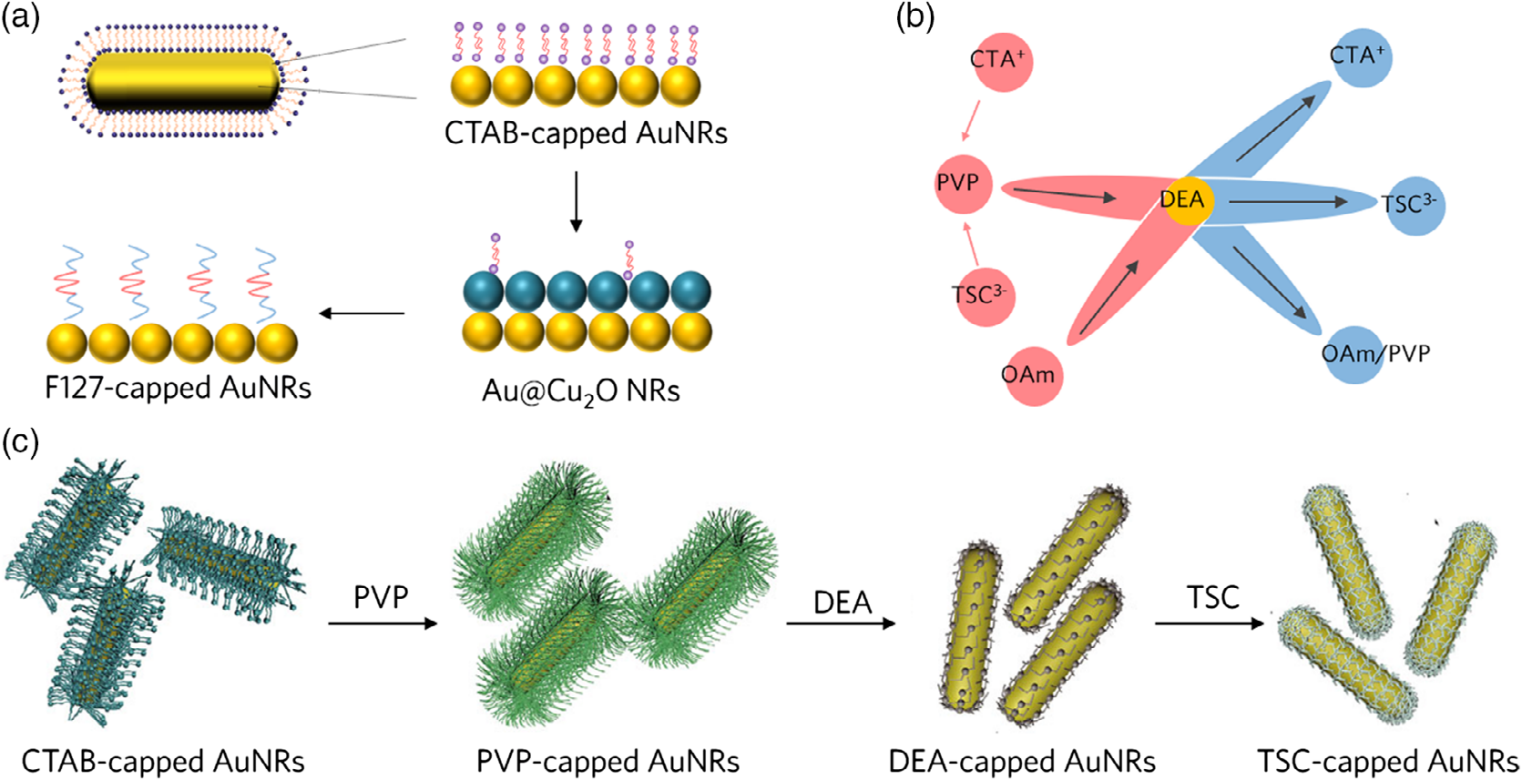
Synthesis of biocompatible plasmonic nanostructures. a) Schematic illustration of indirect capping ligands removal route for AuNRs. Adapted with permission.^[^ ^127^ ^]^ Copyright 2020, Royal Society of Chemistry. b) A diagram for the ligand exchange strategy using DEA as an intermediate capping ligand. c) A typical ligand exchange of AuNRs to replace the native CTAB with TSC. Reproduced under terms of the CC‐BY licence.^[^ ^128^ ^]^ Copyright 2020, The Authors; Exclusive Licensee Science and Technology Review Publishing House.

A universally applicable method is highly desirable to overcome the challenges brought about by non‐biocompatible ligands. Recently, an indirect ligand exchange strategy for effectively replacing toxic ligands on different noble metal nanocrystals (AuNRs, Ag nanodisks, and Pd nanocubes) was demonstrated.^[^ ^126^, ^127^ ^]^ For example, biocompatible AuNRs could be obtained by coating and successive etching of cuprous oxide (Cu_2_O) on their surface (**Figure** **8**a). This strategy is based on the hypothesis that the growth of Cu_2_O on core nanoparticles is quasi‐epitaxial, which excludes the organic ligands on the surface of cores. During the etching process, the probability of the recombination of the original ligand, cetyltrimethylammonium bromide (CTAB), on the nanocrystal surface is neglectable because of the extremely low concentration in the etching solution, making complete ligand exchange possible. The replacement of high‐toxic ligands of CTAB by low‐toxic Pluronic F127 endows AuNRs with very low cytotoxicity to cells.

In general, the weakly bonded ligands cannot replace strong bonded ones, resulting in a limited exchange process. If external stimuli can change the ligand's affinity, it may break the limit of conventional ligand exchange. To this end, we proposed to use diethylamine (DEA) to serve as an intermediate ligand to replace strong capping ligands with weak ones (Figure 8b).^[^ ^128^ ^]^ This process relies on the fact that DEA's binding affinity to the metallic surface can be conveniently switched by controlling the pH. We then used the case of AuNRs as an example to demonstrate the feasibility of our strategy, which is shown in Figure 8c. As DEA has a very strong affinity to Au nanoparticles, it can replace other ligands such as CTAB or PVP. In the presence of trisodium citrate (TSC), the acid protonated the DEA molecules on the Au surface to produce TSC‐capped AuNRs. As a result, the strong capping ligand (CTAB) was successfully replaced by a weak and biocompatible capping ligand (TSC), making these AuNRs suitable for biological applications.

### 3.4 Laser‐Induced Color Printing

**Figure 9:**
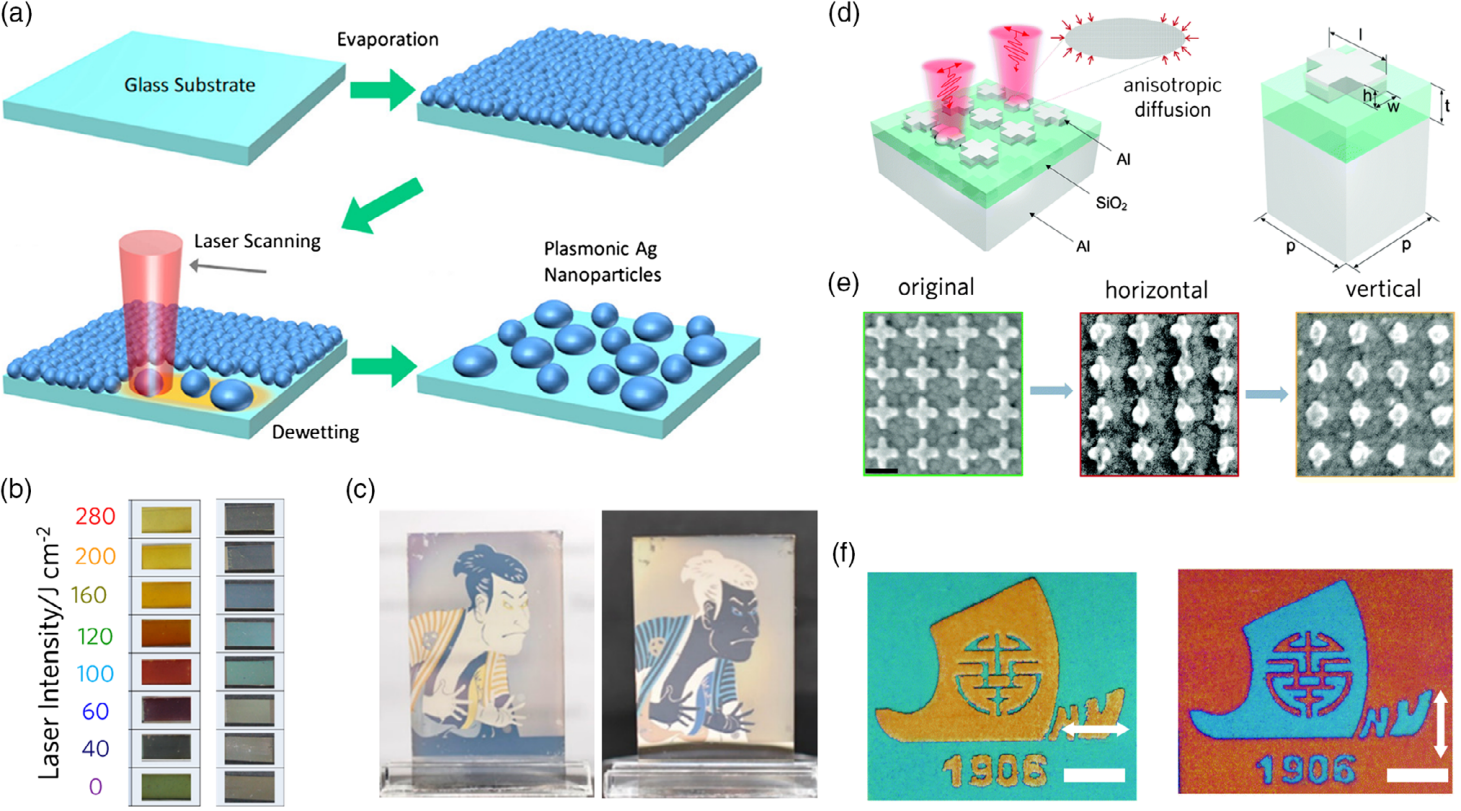
Localized photothermal deformation enabled plasmonic color printing. a) Schematic illustration of laser printing on semi‐continuous Ag films. b) Photographs of printed color with different laser intensities on top of a white (left) and black (right) background. c) Optical image of printed patterns viewed above a white (left) and black (right) background. Adapted with permission.^[^ ^131^ ^]^ Copyright 2020, American Chemical Society. d) Schematic illustration of anisotropic surface diffusion of Al cross structures by polarization‐controlled pulses. e) Top‐view SEM images of 1) initial Al cross arrays, arrays with 2) horizontal arms reshaped first, and 3) then vertical arms reshaped at the laser fluence of 94.6 J m^−2^. Scale bar is150 nm. f) Encrypted color images of two different Jinan University logos with different color appearances. Scale bars are 20 μm. Adapted with permission.^[^ ^136^ ^]^ Copyright 2016, Royal Society of Chemistry.

Because of their pronounced optical response, plasmonic materials have been used for pigmentation for centuries. The stability of the noble metal nanoparticles made them ideal candidates for long‐lasting coloration while dyes degrade over time. With the development of lithography techniques and the application of focused laser, the direct printing of plasmonic colors has been made possible. The localized photothermal effect can heat the particles to several hundred degrees, resulting in the nanoparticles morphing into a spherical shape.^[^ ^129^ ^]^ The photothermal deformation under focused light provided an easy strategy to directly print desired patterns on a substrate. As demonstrated by Kuroiwa and Tatsuma,^[^ ^130^ ^]^ when a semi‐continuous Ag film prepared by sputtering was illuminated by a laser, dewetting of Ag on the surface leads to the formation of nanoparticles because of the heat generated by the photothermal effect (**Figure** **9**a). The size of obtained Ag nanoparticles can be controlled by laser power and illumination time, and various colors can be obtained (Figure 9b). In general, at the same scanning rate, with increasing laser intensity, the Ag film dewetted into smaller particles, resulting a blueshift in the plasmonic peak of the resulting Ag nanoparticles, accompanied with the shift of transmission color from red to yellow. The patterns obtained showed different transmission and scattering colors (Figure 9c), which could be used in security applications. Moreover, the color printing can be achieved inside a polymeric matrix with randomly dispersed Au nanodiscs.^[^ ^131^ ^]^ When illuminated with a laser, the nanodiscs gradually reshaped into spherical particles, and the aspect ratio of the remaining particles could be tuned by controlling the input laser power. Because of the volumetric distribution of the nanodiscs, multiple layers of patterns can be printed within one piece of material.

Despite efforts in direct laser printing of plasmonic nanostructures, the color of plasmonic nanoparticles was dominated by absorption. Therefore, the colors are primarily observed via transmission, and the color saturation is relatively low. For achieving a highly saturated reflection color, plasmonic material‐based metasurfaces consisting of a dielectric layer sandwiched between a plasmonic layer and a metal reflection substrate were fabricated using lithography methods. The reflection peak of a certain metasurface can be tuned by the size of plasmonic pixels and the thickness of the dielectric layer.^[^ ^132^ ^]^ With laser illumination, the top plasmonic layer underwent photothermal deformation, changing the filling factor of the metasurface. It has also been demonstrated that the polarization selectivity of anisotropic plasmonic nanostructures can be coupled into metasurface to achieve printing multiple overlapping patterns in the same plane (Figure 9d).^[^ ^133^ ^]^ In this scheme, Al cross with orthogonal arms was fabricated with a lithography method. The horizontal and vertical arms of the cross only show photothermal deformation when the laser polarization was aligned along with the direction of the arms. The crossed arms provided two printing dimensions distinguishable by the polarization of the reading light (Figure 9e). As a demonstration, the authors printed their university logo in the same layer, which can display two color states when switching between the orthogonal polarizations of the white light illumination (Figure 9f).

### 3.5 High‐Temperature Photothermal Devices

In addition to low‐temperature solar‐thermal applications such as solar steam generation, photothermal materials are also widely used in large‐scale solar energy plants such as solar thermal power generators and solar thermophotovoltaic generators, where the working temperature is too high for most organic light absorbers to last. Moreover, in high‐temperature photothermal applications, the energy loss due to the black body radiation needs to be considered, and spectral selectivity of the photothermal materials becomes essential. The tunable optical properties of plasmonic nanostructures provide opportunities for achieving spectral selectivity, making them promise for high‐temperature photothermal devices.

Despite substantial advances in the synthesis of Au and Ag nanostructures with widely tunable optical properties, the relatively low melting point limited their application in high‐temperature photothermal conversion. The typical working temperature of these devices ranges from 1000 to 1300 °C, exceeding the melting point of bulk Au and Ag. The thermal reshaping of nanoparticles happens at a much lower temperature, making it difficult to maintain the special structures designed for achieving the desired LSPR profiles. As limited by the intrinsic properties of the conventional plasmonic metals, the search for alternative materials is of great importance.

**Figure 10:**
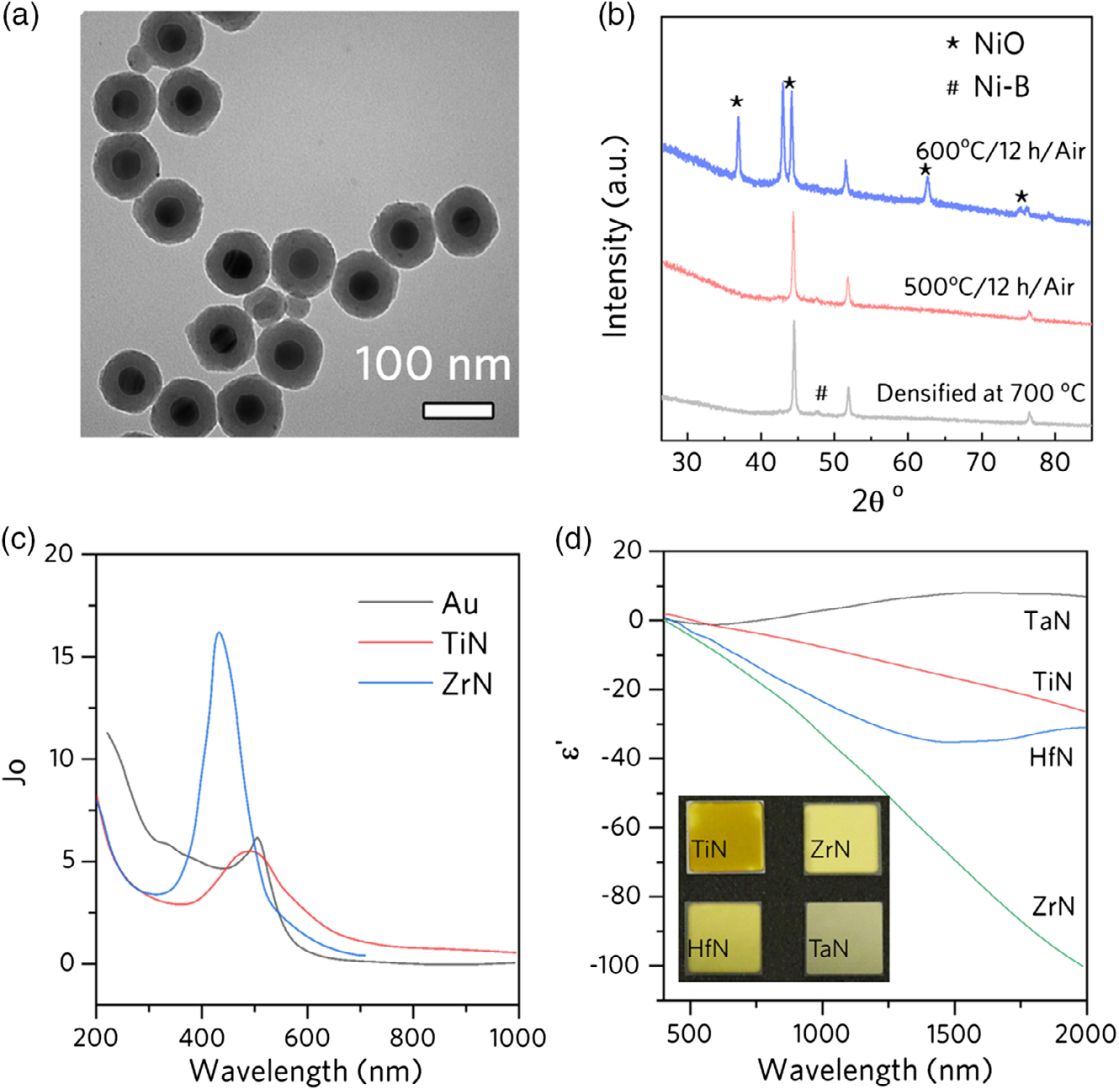
High‐temperature plasmonic nanomaterials. a) TEM images of the Ni@SiO_2_ nanoparticles obtained after annealing at 700 °C. b) X‐ray diffraction patterns of Ni@SiO_2_ nanoparticles densified at 700 °C before and after further annealing at 500 and 600 °C in air for 12 h. Reproduced with permission.^[^ ^139^ ^]^ Copyright 2019, Royal Society of Chemistry. c) Joule numbers of Au, TiN, and ZrN. Reproduced with permission.^[^ ^135^ ^]^ Copyright 2013, Wiley‐VCH. d) Optical image and dielectric functions of metal nitride. Reproduced with permission.^[^ ^140^ ^]^ Copyright 2011, Optical Society of America.

Refractory metals such as tungsten were proposed as plasmonic light absorbers for solar thermophotovoltaic devices due to their high melting point. Also, designs featuring the refractory metals have been reported.^[^ ^134^ ^]^ However, the relatively large imaginary part of the dielectric function limited their photothermal efficiencies.^[^ ^135^ ^]^ Nickle, on the other hand, also exhibits potential in high‐temperature photothermal applications. Although Ni was not typically viewed as strong plasmonic materials, the intrinsically broad absorption band allowed solar energy harvesting without the need for specific structural design. Its high melting point (1455 °C) makes it suitable for solar thermal energy generation. The drawback of the material, however, is the chemical stability. Ni suffers from oxidation, and the plasmonic properties will be compromised by the oxide layer. Unlike aluminum, the oxide layer on the Ni particle surface cannot prevent further oxidation; therefore, the stabilization of nanoparticles is the key to real‐life applications.^[^ ^136^ ^]^ Many methods have been demonstrated to coat the Ni nanoparticles with Al_2_O_3_ or densified SiO_2_ to enhance the stability of Ni nanoparticles against oxidation.^[^ ^137^, ^138^, ^139^ ^]^ As we have demonstrated, Ni nanoparticles produced in colloidal methods were coated with SiO_2_ via the Stöber method (**Figure** **10**a). The high‐temperature annealing of the core–shell nanoparticles in the inert environment increased the condensation degree of SiO_2_, and the densified SiO_2_ proved to be effective in preventing Ni from oxidation up to 500 °C (Figure 10b). Further exploration of the coating materials is believed to significantly promote the applications of Ni as a solar to heat convertor.

In addition to metals, group‐IV metal nitrides, such as TiN, ZrN, and HfN, were considered potential replacements of noble metals due to their high melting point and Au‐like plasmonic properties.^[^ ^140^ ^]^ In a theoretical study conducted by Baffou et al., Joule numbers (*J*_o_) were proposed to evaluate the photothermal efficiency of plasmonic materials based on their dielectric functions. It was pointed out that ZrN holds the potential to outperform Au in the photothermal efficiencies in the visible region, especially at shorter wavelengths (Figure 10c).^[^ ^135^ ^]^ Experimental results also showed the potentials of metal nitrides as plasmonic materials. The sputtered materials showed metallic luster (Figure 10 d), and the negative real part of the dielectric function of TiN, ZrN, and HfN indicated their plasmonic properties. A few designs utilizing metal nitrides as high‐temperature solar‐thermal converter have been demonstrated.^[^ ^141^, ^142^, ^143^ ^]^

## 4 Conclusion and Outlook

With the development of theoretical calculation and synthesis strategies, the focus of the plasmonic photothermal agents is gradually shifting from improving photothermal efficiency to synthesizing nanoparticles with novel structural designs tailored for specific applications. In addition to photothermal conversion, features unique to the plasmonic nanoparticles, such as polarization dependence, catalytic activity, and self‐regulated thermal deformation, are incorporated to bring more complexities and functionalities to the materials.

The key challenges that prevent plasmonic nanomaterials in solar‐to‐heat conversion are the narrow absorption band and the high materials cost. Among many strategies, the plasmonic coupling is the most promising method to broaden the absorption band to match the energy distribution in the solar spectrum. While plasmonic nanostructures with multiple compositions, sizes, and morphologies can be used to broaden absorption, it is equally important to develop methods to organize them in the appropriate configuration to maximize the desired coupling effects. As the fundamental understanding of plasmonic coupling has been understood quite well, theoretical simulation is expected to make a significant contribution to the further development of this field by providing the optimal structural design. More importantly, it is urgent to develop self‐ or directed assembly methods to effectively organize the nanoparticles of complex compositions, sizes, and morphologies into the desired configuration over a large area.

While plasmonic photothermal conversion has its merit for solar energy harvesting, it has intrinsic disadvantages, particularly in the materials cost for large‐scale applications.^[^ ^144^, ^145^, ^146^, ^147^ ^]^ Future development may need to shift the focus of plasmonic materials from noble metals to cheaper ones. For example, alternative plasmonic metals such as Cu and Al should be explored. The challenge is not only in their chemical synthesis into the appropriate size and morphology, but also, more importantly, in their stabilization against oxidation. On the other hand, compared with other photothermal conversion methods, plasmonic heating has its unique advantage of not being constrained by the diffraction limit, making it more suitable for applications requiring localized heating at relatively high temperatures. In addition, localized plasmonic heating can become extremely fast by the excitation of magnetic polariton, reaching a steady state within 1 μs.^[^ ^148^ ^]^ Research in this direction may produce novel devices for applications that require rapid solar heating.

Typical plasmonic photothermal actuators can only perform one predefined deformation in response to a specific wavelength (i.e., ≈520 nm for Au nanospheres). For achieving complex motions, it is required to precisely assemble materials in judicious coordination with the hierarchical structures of actuators during the manufacturing process or periodically change the light source (e.g., light intensity or spatiotemporal distribution) during operation. The inherent complexity in fabrication or operation poses huge challenges for making actuators with multimodal motions. It is believed that by introducing anisotropic magnetic/plasmonic hybrid nanomaterials as photoactive agents, photothermal actuators can be designed to have multimodal motions with the integration of wavelength‐selective and polarization‐selective properties under easily maneuverable light sources. Furthermore, the realization of magnetic control of actuators may extend the potential applications to biomedical fields with multiple response triggers.^[^ ^149^, ^150^ ^]^

The narrow absorption band and localized heat are the most distinctive features of plasmonic properties, which are naturally compatible with single‐wavelength light sources such as lasers. For cancer treatment, the efficient localized heating enabled by plasmonic nanostructures brings significant benefits, making them difficult to be replaced by other photothermal materials; therefore, the use of high‐cost noble metals can be easily justified. The challenge of using plasmonic nanomaterials for biomedical applications is their potential cytotoxicity. Thus, the development of stable and low‐toxic plasmonic nanostructures is particularly important, which, however, is still overlooked in many nanomaterials synthesis laboratories. In addition, with the further development of synthesis strategies, it is possible to incorporate multiple components into a single nanoparticle to enable multifunctionality, providing the potential to build an integrated platform of diagnosis and treatment.

A focused laser brings photothermal printing a high resolution that is hard to achieve using traditional printing techniques. Combining the photothermal effect and strong optical response, plasmonic color printing can achieve vivid color at a resolution near the optical diffraction limit. Meanwhile, the polarization dependence of plasmonic properties allows multiplexed writing and reading modes, making it suitable for information encryption and anticounterfeiting. However, for the intended applications, the relevant technologies remain cost inhibitive at this stage, owing to the reliance on precious metals and low‐throughput fabrication techniques such as e‐beam lithography and focused ion beam milling. In addition, the LSPR peaks in cases of laser‐induced color printing were generally broad due to polydisperse particle size and irregular particle shape resulted from the dewetting process. One possible strategy to achieve a uniform and controllable particle reshaping is the photothermal‐induced fusion of dimer units rather than thermal deformation of randomly distributed structures.^[^ ^151^, ^152^ ^]^ In combination with metasurface construction, a high‐resolution full‐color printing can be achieved. Furthermore, laser‐induced shape morphing is essentially an irreversible process, therefore limiting its application in the dynamic color displays where the colors switch with the exposure to external stimuli. Instead of inducing the deformation of the plasmonic structures, one possibility is to change the dielectric constant of the surrounding through the photothermal effect, which may enable reversible color switching according to Equation (1) and (2).

Spectral selectivity should be emphasized in high‐temperature solar‐thermal conversion applications. The black body radiation needs to be suppressed to minimize energy loss, which, according to Kirchhoff's law of thermal radiation, requires materials to exhibit high absorption in the visible region and a high reflection in the IR region. While the condition cannot be met by the plasmonic materials alone, their combination with other optical structures such as metasurfaces and photonic crystals may provide promising solutions. On the other hand, high‐temperature solar‐thermal conversion applications are again limited by the choices of materials. Although group‐IV metal nitrides have the potential for these applications due to their high stability and metal‐like properties, extensive efforts must be made to develop effective synthetic strategies and incorporate them into the existing device designs.

In recent years, the enhancement of chemical reactions through LSPR has been actively explored in the field of catalysis. The LSPR effect mainly includes local field enhancement, photothermal effect, and hot electron injection.^[^ ^153^, ^154^, ^155^ ^]^ Its vigorous development provides new ideas for breaking the current limitations of photocatalysis and photoelectrocatalysis. For example, research has shown that the integration of plasmonic nanostructures and semiconductors is a promising method to improve solar energy conversion efficiency. However, in most cases, those two are not in full contact, resulting in a limited light conversion. This issue can be addressed by fully embedding metal nanoparticles in semiconductor nanostructures.^[^ ^156^ ^]^ Although LSPR‐enhanced catalysis can couple light energy and heat energy to overcome the energy barrier in the catalysis process, it is still controversial whether the increase in the reaction rate under light comes from the photothermal effect or the hot‐carrier‐induced chemical processes, which calls for more in‐depth mechanism studies.^[^ ^157^ ^]^

We believe that combining physical understanding and materials synthesis is the key to develop plasmonic materials suitable for various photothermal conversion applications. Therefore, the cooperation of researchers with different backgrounds is especially important for designing complex structures at different length scales to solve many of the major challenges we may encounter in realizing these potential applications.

## Conflict of Interest

The authors declare no conflict of interest.
